# Characterization of genetic intratumor heterogeneity in colorectal cancer and matching patient‐derived spheroid cultures

**DOI:** 10.1002/1878-0261.12156

**Published:** 2017-11-27

**Authors:** Sigrid S. Árnadóttir, Maria Jeppesen, Philippe Lamy, Jesper B. Bramsen, Iver Nordentoft, Michael Knudsen, Søren Vang, Mogens R. Madsen, Ole Thastrup, Jacob Thastrup, Claus L. Andersen

**Affiliations:** ^1^ Department of Molecular Medicine Aarhus University Hospital Denmark; ^2^ Digestive Disease Center Bispebjerg Hospital University of Copenhagen Denmark; ^3^ Surgical Research Unit Department of Surgery Herning Regional Hospital Denmark; ^4^ 2cureX Birkerød Denmark; ^5^ Department of Drug Design and Pharmacology University of Copenhagen Denmark

**Keywords:** colorectal cancer, intratumor heterogeneity, patient‐derived spheroid cultures, whole exome sequencing

## Abstract

Patient‐derived *in vitro* cultures of colorectal cancer (CRC) may help guide treatment strategies prior to patient treatment. However, most previous studies have been performed on a single biopsy per tumor. The purpose of this study was to analyze multiple spatially distinct biopsies from CRCs and see how well intratumor heterogeneity (ITH) was recapitulated in matching patient‐derived spheroids. Three to five biopsies were collected from six CRC tumors. Each biopsy was split in two; one half was used for spheroid culturing, while the other half was used for DNA and RNA purification. For two patients, lymph node metastases were analyzed. Somatic mutations were called from whole exome sequencing data. Each tumor contained mutations shared across all biopsies and spheroids, including major CRC drivers such as APC, KRAS, and TP53. At the same time, all tumors exhibited ITH on both mutation and copy number level. The concordance between biopsies and spheroids ranged between 40 and 70% for coding mutations. For three patients, the biopsy and spheroid from matching areas clustered together, meaning that the spheroid resembled the area of origin more than the other areas. However, all biopsies and spheroids contained private mutations. Therefore, multiple cultures from spatially distinct sites of the tumor increase the insight into the genetic profile of the entire tumor. Molecular subtypes were called from RNA sequencing data. When based on transcripts from both cancer and noncancerous cells, the subtypes were largely independent of sampling site. In contrast, subtyping based on cancer cell transcripts alone was dependent on sample site and genetic ITH. In conclusion, all examined CRC tumors showed genetic ITH. Spheroid cultures partly reflected this ITH, and having multiple cultures from distinct tumor sites improved the representation of the genetic tumor subclones. This should be taken into account when establishing patient‐derived models for drug screening.

**Abbreviations**
AF: allele frequency
Cf‐em: cell fraction estimate
CIN: chromosomal instable
CMS: consensus molecular subtypes
CNA: copy number alteration
CRC: colorectal cancer
FFPE: formalin‐fixed and paraffin‐embedded
H&E: hematoxylin and eosin
IGV: Integrative Genomics Viewer
ITH: intratumor heterogeneity
LCM: laser capture microdissection
LNM: lymph node metastasis
LOH: loss of heterozygosity
PDO: patient‐derived organoid
PDX: patient‐derived xenograft
PE: purity estimate
Pt.: patient
SNV: single nucleotide variation
SSC: sessile serrated CRC
WES: whole exome sequencing

## 1 Introduction

Colorectal cancer (CRC) is among the leading causes of cancer‐related deaths in the Western World. In recent years, several studies have documented that CRC is characterized by a considerable intertumor heterogeneity, indicating that it is not a single entity disease (Bramsen *et al*., 2017; De Sousa E Melo *et al*., 2013; Marisa *et al*., 2013; Sadanandam *et al*., 2013). Consistent with this, the existence of four consensus molecular subtypes (CMS) of CRC was recently proposed (Guinney *et al*., 2015) and shown to resolve much of the intertumor molecular heterogeneity. Building on this approach, we recently demonstrated how knowledge of molecular subtypes improves the ability to identify and validate prognostic biomarkers (Bramsen *et al*., 2017), and we foresee that molecular subtyping in the future will lead to improved treatment strategies for CRC. A further complicating factor for molecular subtyping is the intratumor heterogeneity (ITH) that arises during tumor development. After the initial tumorigenic events leading to the malignancy, subclonal mutations are believed to accumulate due to continued genetic instability. These events can be either driver or passenger mutations in relation to tumor evolution. The consequence is co‐existence of genetically distinct subclones within the tumor, potentially with phenotypic differences, for example, in growth, immunogenicity, vascularization, invasiveness, drug response, and metastatic potential (Burrell *et al*., 2013). Personalized treatment strategies, based on drug screens performed on patient‐derived models prior to patient treatment, may be a solution to overcome these issues. Primary models of CRC such as cancer tissue‐originated spheroids and patient‐derived organoids (PDOs) are being established with increasing success rate (Ashley *et al*., 2014; Jeppesen *et al*., 2017; Kondo *et al*., 2011; Sato *et al*., 2011; Schütte *et al*., 2017; van de Wetering *et al*., 2015). These 3D culturing systems increase the success rate of primary cultures and resemble the primary tumor better than traditional one‐dimensional cell culturing (Weiswald *et al*., 2015). Most studies find an overall genetic resemblance between the primary tumor and the established model even after long‐term culturing. Yet, for some patients, they find up to 80% discordant mutations between the primary tumor and the model system (Schütte *et al*., 2017). Most previous studies have been performed with a single biopsy per tumor. Therefore, it is unknown how well the ITH is reflected in the models. There is a need for analyzing multiple cultures per patient to establish how well these models actually represent the genetic ITH of the primary tumor. The aim of this study was to characterize the ITH within CRC and investigate how well it is reflected in matching spheroids derived from multiple spatially distinct sites of the primary tumor.

## 2 Materials and methods

### 2.1 Collection of colorectal cancer tissue samples

**Figure 1:**
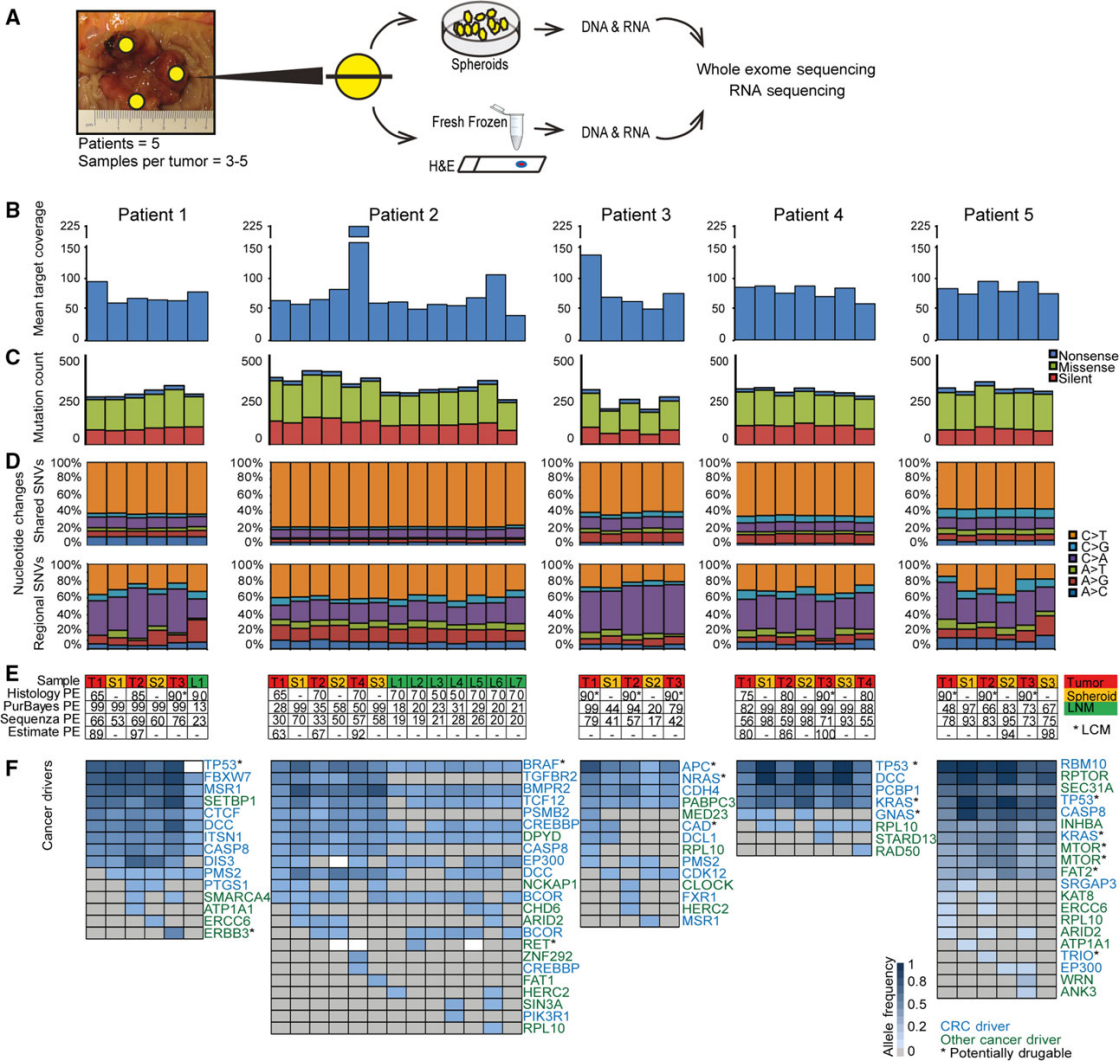
Whole exome sequencing (WES) of multiple primary tumor areas, matched spheroids, and lymph node metastases. (A) Experimental workflow. The biopsies were collected from spatially distinct regions of the tumor (at least 1 cm apart). (B) Mean target coverage of 79X (range 41–225X) from WES. (C) Equal distribution of silent, missense, and nonsense SNVs was observed across samples. (D) Two different mutational patterns were seen when comparing shared SNVs (common for n samples or *n*−1) and regional SNVs (< *n*−1 samples). The pattern observed in the shared SNVs is dominated by C>T mutations in CpG sites, which is a typical age‐related mutational mechanism. (E) Sample information (T = tumor; S = spheroid; L = lymph node metastasis); tumor purity estimates (PE) (%) by Histology, PurBayes (WES data), Sequenza (WES data), and ESTIMATE (RNA sequencing data). *LCM; ‐: not applicable. For most patients, the tumor PEs were higher in the cultures compared to the primary biopsies. (F) CRC drivers (blue) and other tumor drivers (green) were identified through the IntOGen catalog of cancer drivers (blue scale = allele frequency (AF); gray = no calls; white = no calls and < 10 reads). Possible drug targets are marked with an *. In general, mutations in main cancer drivers for CRC (such as TP53, APC, KRAS, DCC, and BRAF) were observed across all samples from each patient. Only one exception from this was observed (TP53 mutation not observed in patient 1_LNM), which was most likely due to too low coverage (< 10 reads). Later‐occurring driver mutations were only present in a subset of the samples from each patient, and furthermore present in lower AFs.

Tumor samples from previously untreated patients were collected at The Surgical Research Unit at Herning Regional Hospital, Denmark. All patients gave written informed consent, and the study was approved by The Central Denmark Region Committees on Health Research Ethics (J. no 1‐10‐72‐221‐14). To assess ITH, three to five tumor regions were biopsied, depending on the largest tumor diameter of 3 cm or 5 cm, respectively (as illustrated in Fig. 1A). The biopsies were collected immediately after surgery (within 30 min). Ischemia times were not registered. Biopsies were taken from the luminal surface from spatially distinct regions of the tumor (east, west, north, south, and from the center). Though a central biopsy was collected only if tumor diameter was > 5 cm. The biopsies were resected by scalpel and were approximately 1 cm*0.5 cm*0.5 cm in size. Each biopsy was divided into two. One half was snap‐frozen in liquid nitrogen and stored at −80 °C for later histochemistry, DNA and RNA purification. The other half was placed in 5 °C transport medium [advanced DMEM/F12 supplemented with 100 U·mL^−1^ Gibco penicillin, 100 μg·mL^−1^ Gibco streptomycin (Life Technologies, Carlsbad, CA, USA), and 2.5 μg·mL^−1^ amphotericin (Sigma‐Aldrich, St. Louis, MO, USA)]. Fresh samples were transported overnight at 5 °C to 2cureX, for the formation of primary spheroid cultures and subsequently DNA and RNA extraction. Six patients were selected for this analysis; however, only four of the patients' tumors were analyzed by both whole exome sequencing (WES) and RNA sequencing. The remaining two tumors were either analyzed by WES or RNA sequencing, respectively. Clinical details are available in Table S1, and a complete sample overview is available in Table S2. Two of the five patients had lymph node metastases (LNMs) at surgery. These were formalin‐fixed and paraffin‐embedded (FFPE) and obtained for DNA extraction.

### 2.2 Laser capture microdissection

From each fresh‐frozen biopsy, a 4‐μm section was cut for hematoxylin and eosin (H&E) staining for a histological overview. Only samples with cancer cell content above 60% were included in the study, to avoid a dominating signal from stromal cells. Biopsies originally presenting with < 60% cancer cells were subjected to laser capture microdissection (LCM) to enrich the cancer cell fraction. For LCM, nine sections of 7 μm were cut, mounted onto Arcturus PEN membrane glass slides (Life Technologies), and stained with Histogene^®^ LCM Frozen Section Staining Kit (Life Technologies) using the manufacturer's protocol, and subsequently stored at −80 °C until LCM. LCM was performed on an Arcturus Veritas 704 (Arcturus Bioscience Inc., Mountain View, CA, USA) and captured on CapSure Macro LCM caps (Thermo Fisher Scientific, Waltham, MA, USA).

### 2.3 Spheroid preparation

Spheroids were established using a modified version (Jeppesen *et al*., 2017) of a previously published protocol (Kondo *et al*., 2011). In brief, the tumor tissue was washed in PBS with antibiotics (500 U·mL^−1^ penicillin, 500 μg·mL^−1^ streptomycin, 100 μg·mL^−1^ gentamicin, and 2.5 μg·mL^−1^ amphotericin B) (Sigma‐Aldrich). Fatty and necrotic areas were cut away with a scalpel, and the tissue was minced into 1‐ to 2‐mm pieces. The minced tissue was washed repeatedly in PBS with antibiotics until the PBS stayed clear. Tissue was digested with 1 mg·mL^−1^ collagenase type II (Life Technologies) in PBS with antibiotics for 30–45 min at 37 °C. The tissue suspension was filtered through a 70‐μm cell strainer (BD Biosciences, San Jose, CA, USA). Retained tissue was redigested for 15–30 min at 37 °C and passed through the filter again. This step was repeated until all tissues passed through the cell strainer. The flow‐through was resuspended in StemPro hESC SFM (Life Technologies) supplemented with antibiotics (200 U·mL^−1^ penicillin, 200 μg·mL^−1^ streptomycin, 100 μg·mL^−1^ gentamicin, and 2.5 μg·mL^−1^ amphotericin B) and seeded in petri dishes coated with a thin layer of 1% agarose (Sigma‐Aldrich) in PBS to avoid cell adherence. Cells were cultured at 37 °C in a 5% CO_2_ humidified incubator for 1–8 days until spheroids had formed. The success rate of establishing cultures was 83%.

For DNA purification, spheroids were washed in PBS to detach debris and loosely attached cells and afterward filtered through a 40‐μm cell strainer. Spheroids retained in the cell strainer were lysed in Cell Lysis Solution with 5 mg·mL^−1^ Puregene Proteinase K (Qiagen, Hilden, Germany) and stored at −80 °C.

### 2.4 DNA and RNA purification

DNA was isolated from fresh‐frozen tissue samples and lysed spheroids using the Gentra Puregene Tissue kit (Qiagen). GenElute‐linear polyacrylamide (Sigma‐Aldrich) was added as a carrier to enhance the outcome. DNA concentrations were quantified using the Qubit dsDNA Broad Range assay (Life Technologies). DNA from FFPE tissue was purified using QIAamp DNA FFPE Tissue Kit (Qiagen). RNA purification was performed using RNeasy mini kit (Qiagen), and RNA quality was measured using Agilent RNA 6000 Nano/Pico Kits on an Agilent 2100 Bioanalyzer (Agilent Technologies, CA, USA). DNA and RNA were stored at −80 °C until analysis.

### 2.5 Whole exome sequencing and data processing

WES was performed using the KAPA‐Hyper prep kit from Illumina (Roche, Basel, Switzerland) for library construction, followed by exome capture using NimbleGen SeqCap EZ Human Exome Library v3.0 (Roche). Sequencing was performed on an Illumina NextSeq500, with 10–50 ng of genomic DNA as input per sample (depending on material available). Reads were mapped using BWA MEM against the human reference genome HG19, and duplicates were marked with Picard MarkDuplicates. SNPs were called using GATK HaplotypeCaller. Somatic mutations were called using MuTect2, with matched germline WES data obtained from blood samples as reference. Mutation allele frequencies (AFs) were calculated using SAMtools mpileup. Copy number alterations (CNAs) and allelic imbalance were estimated using FACETS (Shen and Seshan, 2016). Tumor purity was estimated using PurBayes (Larson and Fridley, 2013) and Sequenza (Favero *et al*., 2015). Tumor drivers and potentially druggable targets were identified using the IntOGen catalog of cancer drivers, available for download at Intogen.org (Tamborero D, Rubio‐Perez C, Deu‐Pons J, Schroeder M, Vivancos A, Rovira A, Tusquets I, Albanell J, Rodon J, Tabernero J, Dienstmann R, Gonzalez‐Perez A and Lopez‐Bigas N, unpublished data).

### 2.6 Phylogenetic analysis and heatmaps

Phylogenetic trees were generated as previously described (Thomsen *et al*., 2016). Shortly, we used the presence/absence of each single mutation to score each possible rooted phylogenetic tree and the highest scoring tree was used. The length of the branches is proportional to the number of mutations supporting this separation. The origin of the tree is the ancestral clone where the first mutation occurred.

Heatmaps were created using the function *aheatmap* from the R package, *NFM*, with ‘binary’ distance and the linkage method ‘average’. All somatic single nucleotide variations (SNVs; missense, nonsense, and silent) supported by two or more reads in a given sample, and absent in the matched germline (0–1 read), were included. To avoid false negatives, regional mutations with less than ten reads in negative samples were not included in the heatmaps.

### 2.7 RNA sequencing and data processing

RNA sequencing was performed as previously described using ScriptSeq RNA‐Seq Library preparation Kit from Illumina (Hedegaard *et al*., 2016). The paired raw sequence reads were processed using TopHat2 (Kim *et al*., 2013) and mapped to the human reference genome HG19. FPKM values were called using Cufflinks (Trapnell *et al*., 2010) and GenCode v19 transcript information. The FPKM gene expression values were used to assign a molecular subtype to each sample using our previously reported TUMOR subtype classifier (Bramsen *et al*., 2017). CMS were assigned using the nearest‐centroid Single Sample Predictor CMS classifier (Guinney *et al*., 2015), and CRIS types were assigned using the CRIS classifier (Isella *et al*., 2017). Tumor purity was estimated from the RNA sequencing data using ESTIMATE (Yoshihara *et al*., 2013).

### 2.8 Data availability

WES and RNA sequencing data are available via European Genome‐phenome Archive (EGA) under EGA study ID EGAS00001002684. The sample IDs submitted to the EGA are available in Table S2.

## 3 Results

### 3.1 Genomic characterization of multiple primary tumor areas and matched spheroids

We performed multiregional WES on tumor material from five CRC patients. Each tumor was biopsied at three to five spatially distinct sites, and each biopsy was divided into two. One half was dissociated and grown as spheroid culture, while the other half was snap‐frozen (Fig. 1A). For two patients, regional LNMs were identified during pathological examination of the resected tumor specimen. The cancer percentage of each biopsy was assessed by histology, and all samples with a cancer percentage below 60% were purified by LCM before DNA and RNA extraction. DNA from tumor biopsies, spheroids, and LNMs were analyzed by WES. The mean target coverage obtained was 79X (range 41–225X) (Fig. 1B). A similar distribution of silent, missense, and nonsense SNVs was observed across samples (Fig. 1C). For all patients, two different mutational patterns were observed when comparing shared mutations (common for *n* or *n*−1 samples) to regional mutations (common for < *n*−1 samples) (Fig. 1D). C>T mutations were dominant in the shared mutations and occurred predominantly in a CpG site context (*P* < 2.2e‐16, Pearson's chi‐squared test), consistent with an age‐related mutational mechanism (Milholland *et al*., 2015). Regional mutations were characterized with a lower frequency of C>T mutations and in some patients, an increase in C>A mutations. We estimated the cancer cell purity of each sample using a variety of methods: histology, WES data (PurBayes and Sequenza), and RNA sequencing data (ESTIMATE). The purity estimates ranged between 13 and 100% among the samples. The concordance between the different methods was low, although they followed the same tendencies (Fig. 1E). Generally, the spheroids were estimated to be purer than the matching tumor biopsies (*P* = 0.02, paired Wilcoxon signed rank test) (Fig. 1E). Analysis of known cancer driver genes, as defined by IntOGen (Tamborero D, Rubio‐Perez C, Deu‐Pons J, Schroeder M, Vivancos A, Rovira A, Tusquets I, Albanell J, Rodon J, Tabernero J, Dienstmann R, Gonzalez‐Perez A and Lopez‐Bigas N, unpublished data), revealed a striking mutational pattern. For each patient, the shared driver genes were significantly enriched for genes known to drive CRC pathogenesis, such as APC, KRAS, BRAF, DCC, and TP53. By contrast, the regional driver genes were enriched for genes without a strong link to CRC (*P* = 0.0006, Pearson's chi‐squared test). Furthermore, the variant AFs of regional drivers were typically lower, indicating that the mutations were subclonal within the sample (Fig. 1F). Cancer drivers are obvious drug targets. Therefore, we investigated whether potentially druggable drivers were present in all samples or only in a subset. Possible drug targets were identified using the IntOGen database; some were shared, while others were regional (Fig. 1F). Mutations in potentially druggable genes were shared across all samples for each patient in 11 of 27 events, and all patients had at least one shared mutation in a potentially druggable gene. For example, BRAF was mutated in all tumor, spheroid, and LNM samples of patient 2 (pt. 2), and therefore, a target that would likely allow the majority of pt. 2′s cancer cells to be targeted.

### 3.2 Genetic intratumor heterogeneity in the primary tumor and lymph node metastases

**Figure 2:**
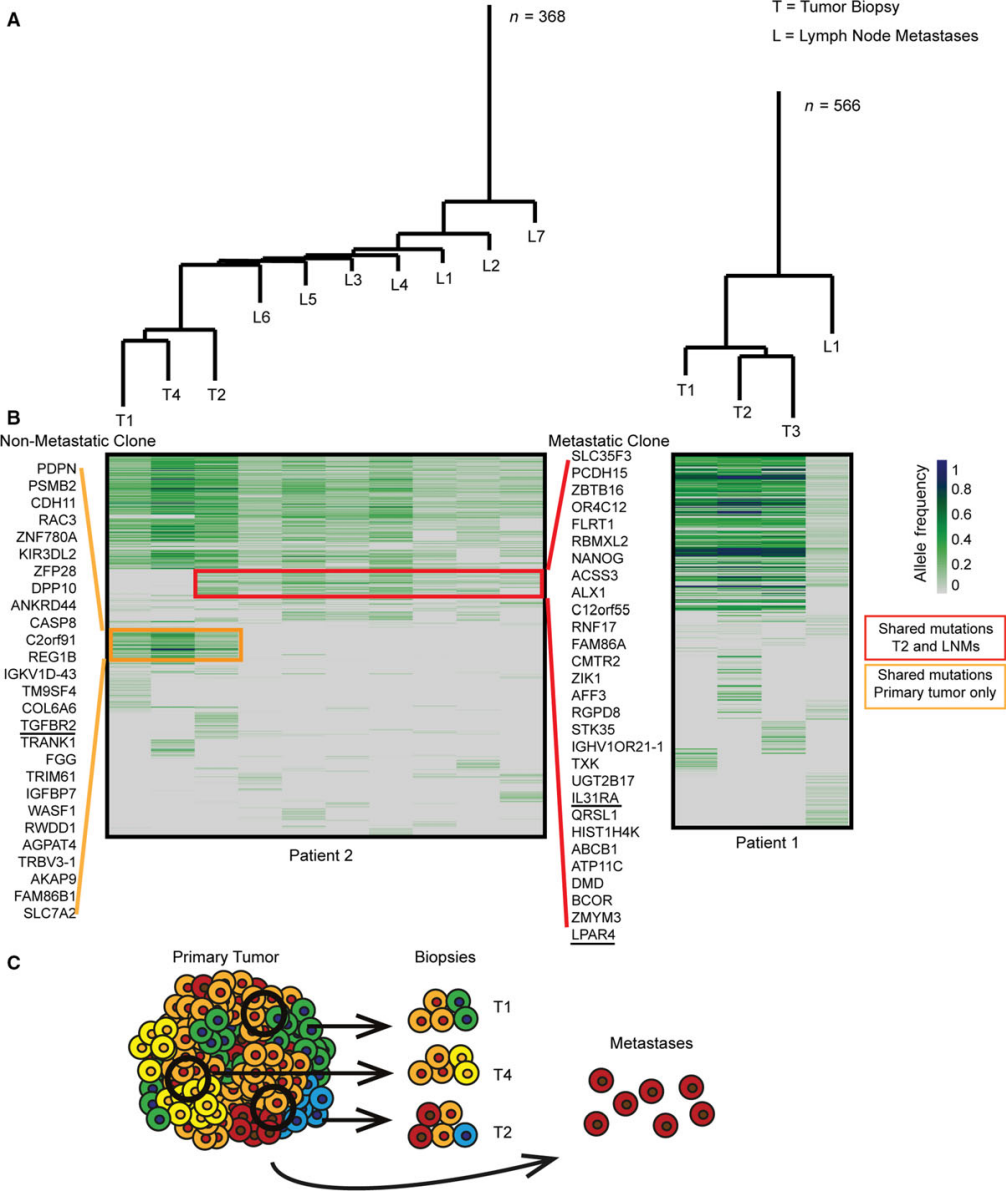
Genetic intratumor heterogeneity in primary tumor and lymph node metastases. Phylogenetic trees and heatmaps based on all mutations from each sample (silent, missense, and nonsense). (A) Phylogenetic trees. Patient 2, the seven LNMs cluster together indicating a common origin. Patient 1, tumor areas cluster together; however, each area contains multiple private mutations. The length of the branches corresponds to the number of mutations separating the samples; n‐value indicates the total number of mutations. (B) Heatmaps illustrating allele frequency (AF) of SNVs (green/blue scale). Regional mutations were excluded if < 10 reads in uncalled samples. A common ancestral block of mutations was observed for both patients. Patient 2, the red box marks the mutations only called in the metastatic clone (present in all LNMs but only in tumor area 2 of the primary tumor). The orange box marks the mutations that are only called in the nonmetastatic cell clone (present in all tumor areas, but not in the LNMs). Patient 1, LNMs have no clear connection to a certain area of the tumor. (C) Illustration of genetic subclones in primary tumor biopsies and metastases in patient 2. The metastatic clone is only found in one of three biopsies and hence not the dominant clone in the primary tumor.

Two patients in this study were stage III presenting with local LNMs. To compare the genetic heterogeneity within tumors and their LNMs, phylogenetic trees and heatmaps were created based on all mutations identified in each sample (silent, missense, and nonsense). For both patients, the trees indicated that seeding of the LNMs had happened from a clone ancestral to those currently found in the tumor. After seeding of the metastases, the clones evolved in separate directions in the LNMs and in the tumor (Fig. 2). For pt. 1, one LNM was analyzed and its mutational pattern was equally consistent with the patterns observed in either of the tumor areas. Accordingly, it cannot be determined from which tumor area the cell seeding the metastasis originated (Fig. 2A,B). For pt. 2, seven distinct LNMs were analyzed. The phylogenetic tree indicated a very close relationship between them (Fig. 2A), suggesting that they probably all originated from the same original clone. Furthermore, this clone most likely originated from tumor area 2 (T2), as T2 is closest to the LNMs in the phylogenetic tree (Fig. 2A). Consistent with this, the mutational pattern of the LNMs is observed in T2, but not in the two other tumor areas (T1 and T4) (Fig. 2B). We also noticed a set of mutations common for all tumor areas, but not seen in the LNMs (Fig. 2B), indicating that they originate from a nonmetastatic subclone. If we had not analyzed the LNMs, these mutations would most likely have been interpreted as ancestral. Taken together, our data indicate that at the time of metastatic seeding at least two distinct clones co‐existed in the tumor. It is worth noticing that at the time of diagnosis, the metastatic clone only constituted a minor fraction of the primary tumor, being found in only one of three tumor regions examined, as modeled in Fig. 2C. Meaning that by analysis of a single biopsy from the tumor, critical information about the metastatic clone could easily have been missed. Interestingly, the nonmetastatic clone had a mutation in TGFBR‐II, which is known to reduce the metastatic potential (Fig. 2B) (Armaghany *et al*., 2012). The metastatic clone, on the other hand, had several mutations associated with increased metastatic potential. These included an inactivating (nonsense) mutation in the metastasis inhibiting IL31 receptor gene (IL31RA) (Davidi *et al*., 2017), and a missense mutation in lysophosphatidic acid receptor 4 (LPAR4). LPAR receptors 1‐6 have been implicated in various prometastatic functions in different cancer types (Willier *et al*., 2013). However, LPAR4 seems to have an antagonistic motility impact on CRC cells (Lee *et al*., 2008), and a recent study showed that knockdown of LPAR4 increased motility of CRC cells (Takahashi *et al*., 2017).

### 3.3 Genetic intratumor heterogeneity in the primary tumor and spheroids

**Figure 3:**
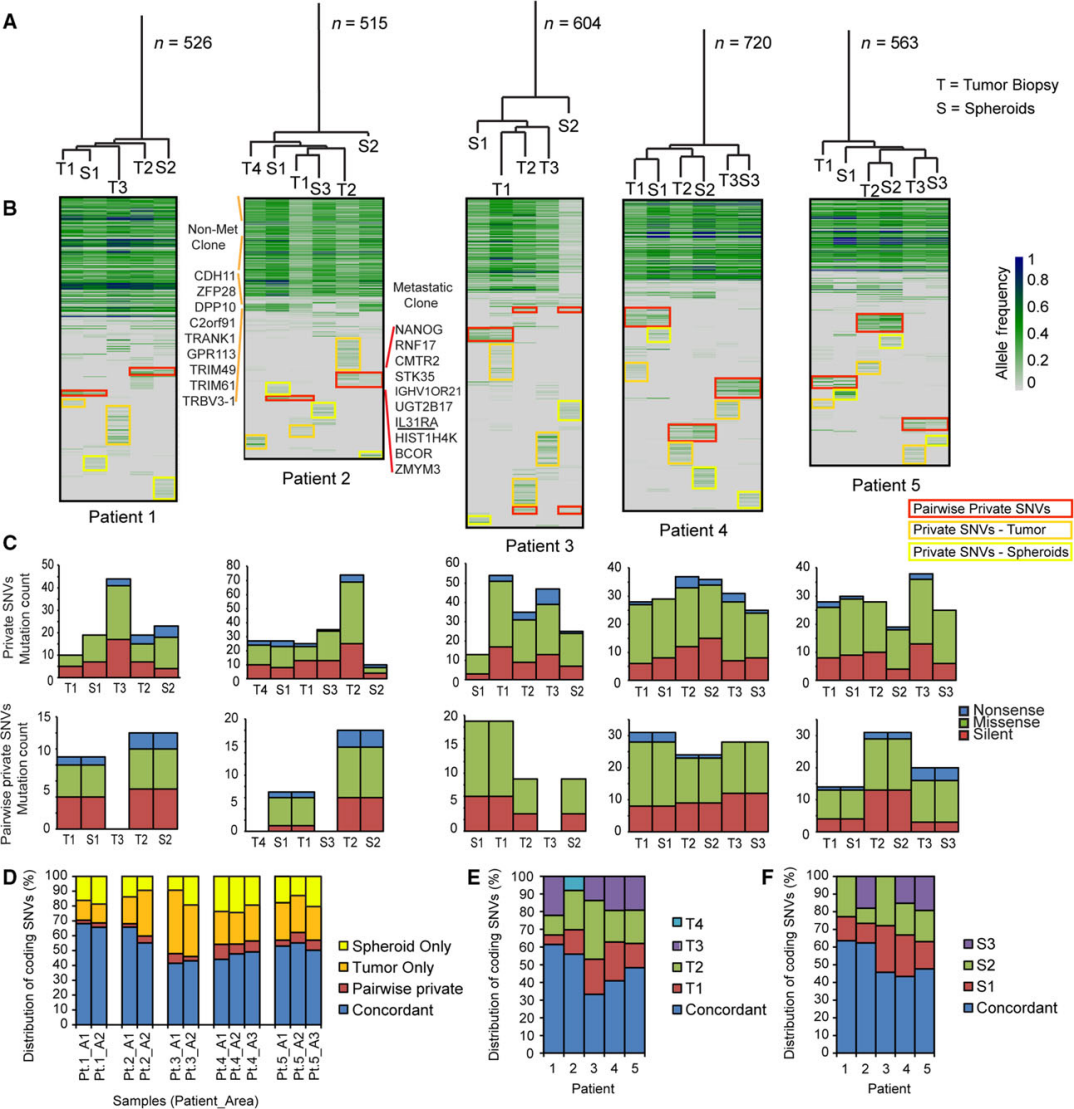
Genetic intratumor heterogeneity in primary tumor and spheroids. (A) Phylogenetic trees based on all called SNVs from primary tumor biopsies and spheroid cultures. The length of the branches corresponds to the number of mutations separating the samples; *n*‐value indicates the total number of mutations (T = tumor; S = spheroid). (B) Heatmaps based on all SNVs. Green/blue scale indicates AF. A large block of shared mutations are common for all patients. Nevertheless, all samples have private mutations (yellow and orange boxes) and pairwise private mutations between tumor biopsy and matched spheroid (red boxes). For patient 2, the mutations characterizing the nonmetastatic and the metastatic clones (given in Fig. 2) are indicated on left and right side of the heatmap. Nearly all mutations from the nonmetastatic clone were present in all samples. Only in S3 a few mutations were undetected. These are listed. The mutations specific to the metastatic clone were primarily observed in T2, although a few were also seen in S2 at low AFs. (C) Private and pairwise private mutations are a mix of silent, missense, and nonsense mutations. (D) Distribution of coding SNVs (%) comparing tumor only, spheroid only, pairwise private, and concordant mutations for each area. (E) Distribution of coding SNVs (%) comparing all tumor biopsies for each patient. The degree of ITH varies between patients. (F) Distribution of coding SNVs (%) comparing all spheroid cultures from each patient.

Spheroid cultures have been suggested as *in vitro* models of primary cancers. To address how well spheroid cultures reflect the genetic ITH observed in primary CRC tumors, matched spheroids and biopsies from multiple tumor regions were analyzed. The phylogenetic trees (Fig. 3A) and mutational heatmaps (Fig. 3B) were generated based on nonsense, missense, and silent SNVs called from WES profiles. A long ancestral trunk with shared mutations across all samples was common for all patients (Fig. 3A). When comparing the distance (number of mutations distinguishing samples in the phylogenetic tree) between matching tumor biopsy and spheroid pairs, there was a greater similarity between the mutational profile of matched tumor biopsies and spheroid pairs than between biopsies from different areas in the tumor (*P* = 0.03, Wilcoxon rank sum test). However, this could simply be due to less heterogeneity in the spheroids and thereby shorter private branches. For some patients (pt. 1, 4, and 5), the matched tumor biopsies and spheroids clustered together. However, for others (pt. 2 and 3), there was no clear connection between the matched tumor biopsies and spheroids. For pt. 3, the tumor samples cluster together, indicating dissimilarity between the matched spheroids and tumor regions (Fig. 3A). Patient 3 also stands out by having lower AFs in the spheroid cultures, particularly spheroid 2, than in the matching biopsies (Fig. 3B). This could indicate that the spheroid cultures contained nonmutated cells, potentially adenoma cells, which might also explain the unusual clustering. For most patients, the AFs in spheroids were higher than the primary biopsies (Fig. 3B), indicating that spheroids generally had a higher cancer cell content than the biopsies.

Notably, all samples had private mutations, which constituted on average 10% of the observed mutations (range 3–18%). Pairwise private mutations observed only in the biopsy and spheroid from the same location were observed across all patients but to varying degrees [on average, they constitute 6% of all mutations (range 2–10%)]. For pt. 2, the mutations present in the metastatic and nonmetastatic clones (illustrated in Fig. 2B) were also present in the spheroids (Fig. 3B). The mutations from the nonmetastatic clone were present in all spheroid cultures. The metastatic clone, on the other hand, was only evident in spheroid culture 2, consistent with this clone being identified only in biopsy 2 (Figs 2 and 3B). However, only some of the mutations were called and these were present in very low AFs. This indicates that the metastatic clone was a minor clone in the spheroid 2 culture; hence, uncalled mutations from the metastatic clone might be false negatives.

Both the private and pairwise private mutations from the biopsies and the spheroids were a mix of silent, missense, and nonsense mutations (Fig. 3C). When looking only at the coding SNVs (Fig. 3D), the concordance between the biopsy and spheroid from each area varied from 40 to 70%. The pairwise private SNVs represent a minor fraction (mean 5%), while the SNVs private for either the primary tumor biopsy (mean 24%) or spheroids (mean 17%) constitute a larger part. Looking only at the ITH of coding SNVs in the primary tumor (Fig. 3E), it is clear that the level of ITH varied between patients. This variation was mimicked in the spheroid cultures (Fig. 3F), indicating that high ITH is associated with high divergence between spheroid cultures.

### 3.4 Selection for tumor driver mutations through CNAs

**Figure 4:**
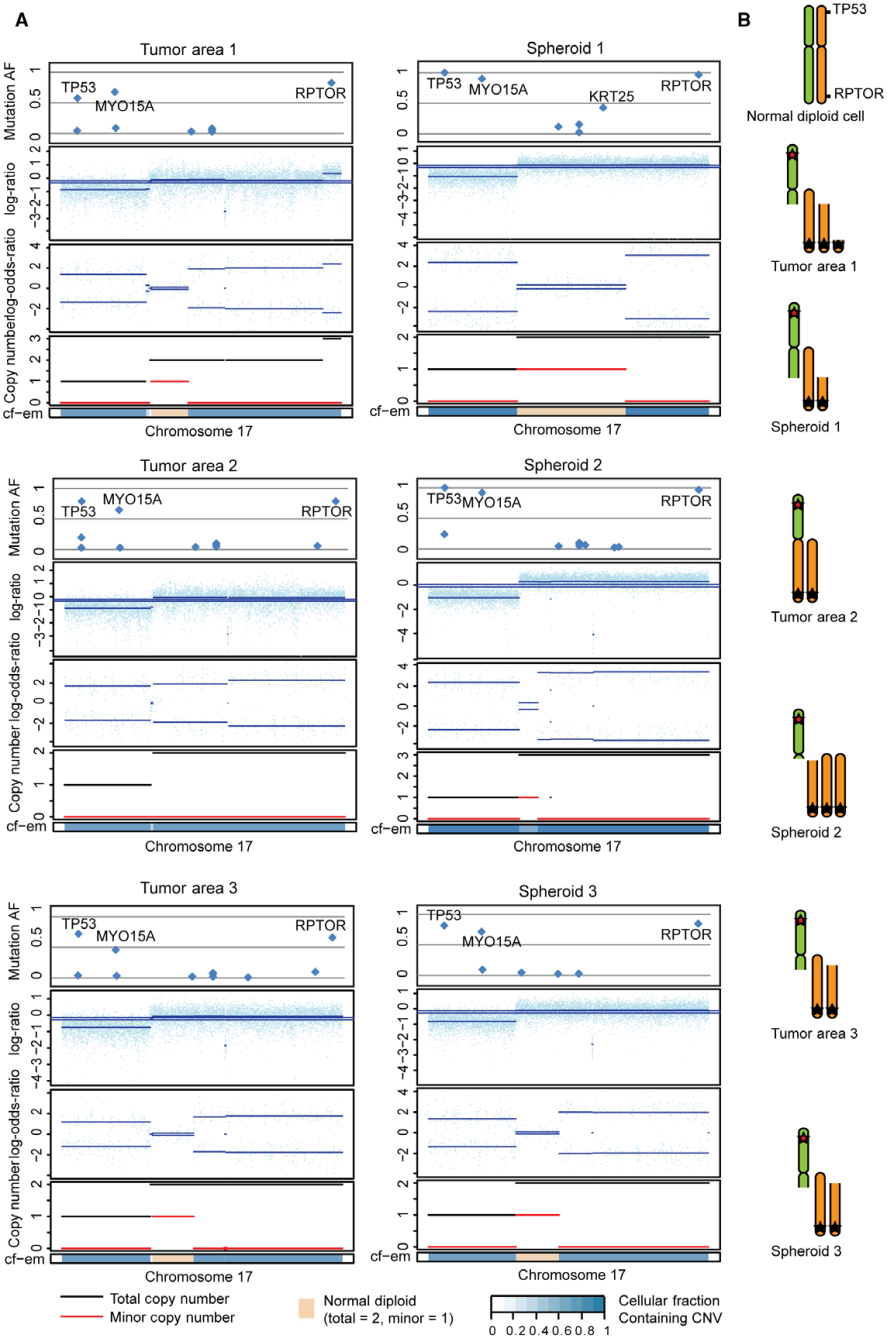
Parallel evolution: selection of different clones with multiple copies of mutated RPTOR obtained through different chromosomal alterations leading to copy number alterations (CNAs). (A) CNAs for chr.17 are plotted for all samples from patient 5. CNA analysis of the exomes using FACETS revealed variations in CNAs between samples. Mutational AF, relation between minor and major allele, copy number, and cell fraction estimate (cf‐em) are plotted. (B) Illustration of chr. 17 for all samples from patient 5. LOH of 17p‐arm (incl. TP53) and uniparental disomy or trisomy of 17q‐arm with mutated RPTOR. Different fractions of the 17q‐arm (with the mutated RPTOR) are gained, which indicates that the mutation occurred prior to the CNAs and that multiple copies of the mutated RPTOR give an advantage.

In addition to point mutations, allelic imbalances and CNAs were also called from the exomes using the tool FACETS (Shen and Seshan, 2016). For each patient, the analysis revealed an extensive level of structural genomic variation between regional biopsies and spheroids. For example for pt. 5, differences in CNAs were observed in several areas of nearly all chromosomes. In general, the CNAs detected in the spheroid cultures had higher amplitudes and were more uniform than in the matching primary biopsy, indicating that a larger fraction of the cells in spheroids contained the CNA (e.g., chr.3, Fig. S1). In Fig. 4A, the CNAs of chr.17 are plotted for each sample from pt. 5. Common for all samples are loss of the short arm of chr.17, leading to loss of heterozygosity (LOH) at the TP53 locus. In contrast, each biopsy and spheroid culture showed a near unique copy number pattern for the long arm of chr.17. Some samples show LOH along the whole long arm, while in others, only parts of the long arm are affected. Often the retained allele is duplicated or triplicated (fully or partially) leading to regions with uniparental disomy and trisomy (illustrated in Fig. 4B). A region close to the telomere‐end of the q‐arm stands out. It contains a missense mutation in the RPTOR gene with an AF close to one in many samples. The high AF combined with multiple different amplification patterns of the region suggests that the mutation occurred prior to the CNAs (Fig. 4A). Of the observed q‐arm mutations, only the RPTOR mutation reached an AF close to one, indicating that only the RPTOR mutation was present prior to the CNAs. RPTOR, together with mTOR, is a part of the mTORC1 complex, which is known to play a major role in carcinogenic cell signaling (Kim *et al*., 2017; Wang and Zhang, 2014). RNA sequencing data from spheroids S2 and S3 showed that only the mutated RPTOR allele was expressed consistent with the other allele being lost (Fig. S2A). Taken together, this indicates that the mutated RPTOR may create a selective advantage and play an important role in the tumor progression, which in this case have led to parallel evolution on CNA level resulting in different copy number gains of the mutated RPTOR. To test this hypothesis, we used cBioPortal.org (Cerami *et al*., 2012; Gao *et al*., 2013) to analyze the overall survival of CRC patients with and without RPTOR mutations using a provisional TCGA cohort (*n* = 633) (Fig. S2B) (Available at: http://bit.ly/2tbMjVk). These data indicate that mutations in RPTOR on either SNV or CNA level may be associated with a lower survival, which supports the possible tumor driving function of RPTOR.

### 3.5 Classification of molecular subtypes

**Figure 5:**
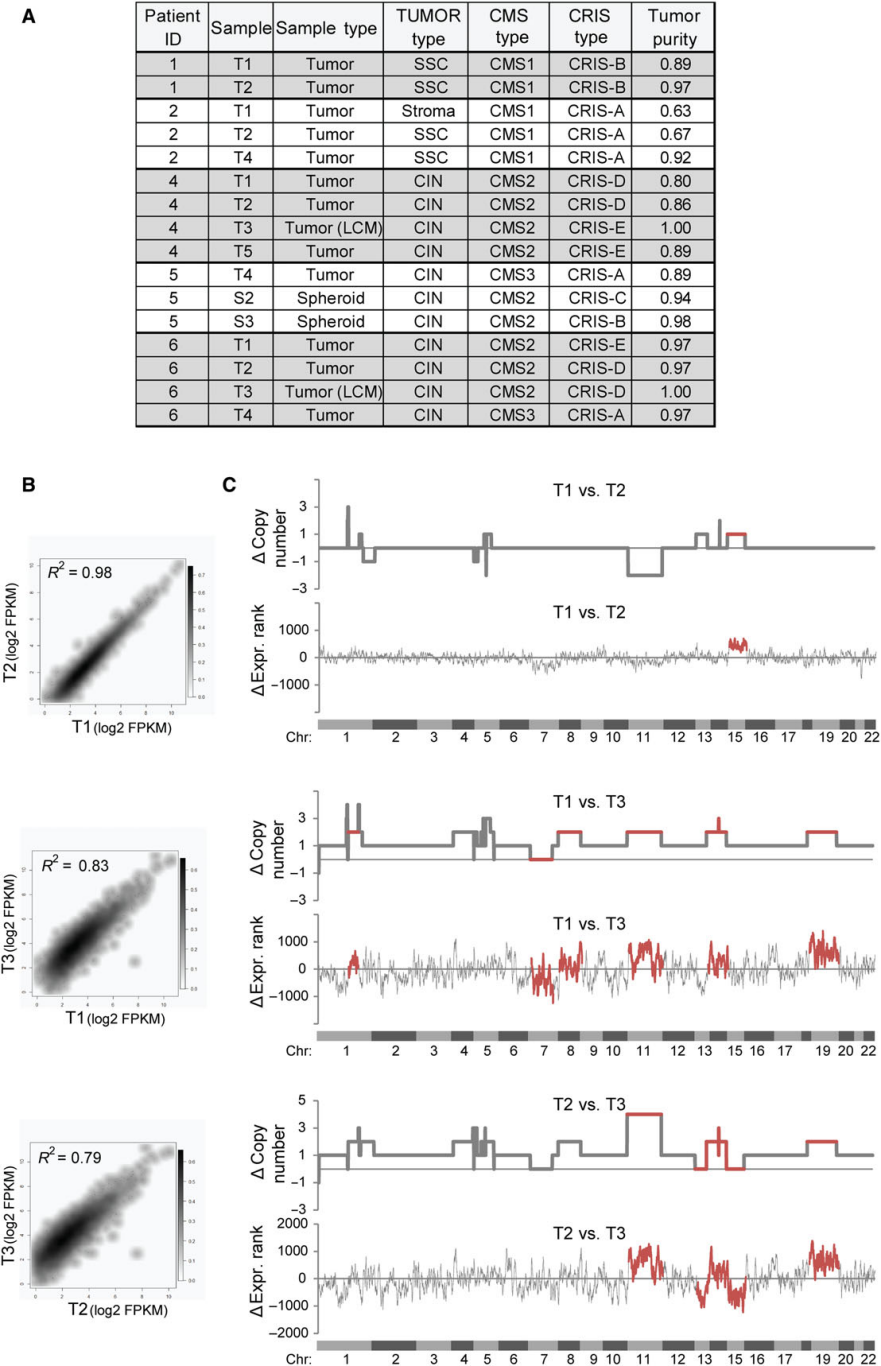
Intratumor heterogeneity as evaluated by RNA sequencing. (A) RNA sequencing‐based classification of tumor samples into molecular TUMOR subtypes (Bramsen *et al*., 2017), consensus molecular subtypes (CMS) (Guinney *et al*., 2015), and CRIS subtypes (Isella *et al*., 2017). ‘Sample’ indicates the tumor/spheroid area, while ‘Sample Type’ indicates the origin of RNA. Tumor purity was evaluated by the estimate software (Yoshihara *et al*., 2013). (B) Density scatter plot showing the correlation in RNA expression between biopsy sites T1–3 from patient 4 for cancer cell‐associated transcripts (i.e., transcripts were only included if FPKM > 5 and if they were of epithelial origin as devised by Isella *et al*. (2017)). (C) Line charts showing the differences in chromosomal copy numbers (as evaluated by FACETS) and gene expression rank changes (‘ΔExpr Rank’) for the transcripts included in (B) between patient 4 tumor biopsies T1–T3 along the human genome. The analysis indicates several regions where both copy numbers and gene expression changes differ between biopsy sites (highlighted in red), but also that T1 and T2 have a higher overall ploidy (triploid) than T3 (diploid). Chromosomal locations are indicated.

Recently, it has been shown that CRC consists of several molecularly and clinically distinct tumor subtypes (Bramsen *et al*., 2017; Guinney *et al*., 2015), which are defined by both the cancer cells and surrounding stromal and immune cells (Bramsen *et al*., 2017). To investigate whether ITH influences classification of CRC tumors into tumor subtypes, we performed RNA sequencing and extracted transcriptional profiles for the biopsies and spheroids from which sufficient material was available (sample overview available in Table S2). We assigned molecular subtypes to each sample using our previously reported TUMOR subtype classifier (Bramsen *et al*., 2017), the CMS classifier (Guinney *et al*., 2015), and the cancer cell CRIS classifier (Isella *et al*., 2017). The latter reportedly only includes epithelial transcripts and reports on the cancer cell subtype, while the TUMOR and CMS classifiers include both epithelial and nonepithelial transcripts (stromal and immune cell transcripts) and reports on overall tumor subtype. Nearly all biopsies from the same patient were classified with the same TUMOR (1 of 16 biopsies with deviant subtype) and CMS subtype (2 of 16 biopsies with deviant subtype) (Fig. 5A). By contrast, classification according to the CRIS subtypes indicated a higher degree of transcriptional ITH at the cancer cell level (7 of 16 biopsies with deviant subtype, Fig. 5A), which supports the genetic ITH observed by WES (Fig. 3).

Taken together, this suggests that while the tumor cell type composition, as evaluated by the TUMOR and CMS subtypes, is largely similar between biopsies from the same tumor, the cancer cells within each biopsy can be molecularly different. The biopsies from patient 4 illustrate this point. Both the TUMOR and CMS classifiers assigned the same subtype to all three biopsies (T1–3), while the CRIS classifier indicated that at the transcriptional level the cancer cells in biopsy T3 were different from those in T1 and T2 (Fig. 5A). Correlation analysis based on FPKM levels of epithelial‐derived transcripts supported this finding (Fig. 5B). Also genetically, the cancer cells of T3 differed from those of T1 and T2 (Fig. 5C). At the genome‐wide level, the gross changes in DNA copy number levels were mimicked in RNA expression profiles (Fig. 5C), suggesting that the assignment of the CRIS subtypes may be driven indirectly by genetic ITH.

It has been reported that tumor subclassification systems based on both epithelial and nonepithelial transcripts could show a variation depending on sampling site, which is primarily due to differences in the fraction of nonepithelial cells in the biopsies (Dunne *et al*., 2016). However, the few samples with deviant TUMOR and CMS subtype assignments in our study had tumor purities that were indifferent from the other biopsies, indicating that this was not the explanation here. Therefore, we speculate that deviant biopsies have a different composition of nonepithelial cell types than the other biopsies, for example, a shift in the stromal‐to‐immune cell ratio.

## 4 Discussion

We performed WES on primary CRC biopsies and matching spheroid cultures derived from multiple spatially distinct sites of each tumor from five patients. Furthermore, we included WES analysis of LNMs for two of the patients. We found spatial genetic ITH within all the primary tumors on both SNV and CNA levels. Well‐known early tumor drivers such as APC, TP53, and KRAS were shared among all samples in concordance with previous studies of ITH in CRC (Hardiman *et al*., 2016; Kim *et al*., 2015). The mutation events in other driver genes were often regional and subclonal with lower AFs (Fig. 1F). Interestingly, we found that the mutational pattern of the metastatic clone in one patient with seven LNMs was only found in one of three regions in the primary tumor (Fig. 2). This means that if only a single biopsy of the primary tumor had been analyzed, the metastatic clone could easily have been missed. As the standard in the clinic today is mutational analysis of a single biopsy, this could easily have been the case. Multiple biopsies from spatially distinct sites of the tumor improve the chance of identifying ancestral targets. However, in this case, not all the mutations shared between the primary tumor biopsies were observed in the LNMs. Without the information about the LNMs, these mutations would have appeared to be ancestral. At the same time, the mutations present in the metastatic clone would appear as private for one of the areas, and therefore as less important targets. This underlines the complications that ITH brings for both biomarker development and treatments strategies.

Patient‐derived 3D tumor models may be used to perform drug screens prior to patient treatment. However, it is unclear how well these models recapitulate the ITH of the primary tumor. Unlike most previous studies of patient‐derived models (Schütte *et al*., 2017; van de Wetering *et al*., 2015), we established multiple spheroid cultures from distinct sites of each tumor. This approach allowed us to investigate the spatial ITH of CRC and how well it was reflected in the spheroids. Each spheroid culture resembled the area of origin more than other tumor areas. This indicates that the spatial genetic ITH in the tumor was reflected in the spheroids. Furthermore, the AFs for the SNVs and the amplitudes of the CNAs were typically larger in the spheroids, indicating a higher cancer cell purity.

We detected private mutations in both primary biopsies and spheroid cultures (Fig. 3). The private mutations in the tumor biopsies indicate that not all subclones are represented in the spheroids. On the other hand, the private mutations in the spheroids are most likely observed due to a selection of rare subclones, which increases the detection level compared to the primary tumor. As the primary tumor biopsies generally have lower tumor purities than the spheroid cultures, it cannot be excluded that increasing their read depth would also increase the overlap in identified mutations. The spheroids were only short‐term‐cultured, less than 8 days; hence, we find it unlikely that the private mutations were acquired during culture. Another explanation could be that even though the tumor cells used for sequencing and for spheroid formation originated from the same biopsy, they were nevertheless not identical due to local ITH. In a recent study by the OncoTrack consortium, both PDO and patient‐derived xenografts (PDX) cultures were established from a large number of patients (Schütte *et al*., 2017). They established PDO models from a single biopsy per tumor. For some patients, sibling pairs of PDO and PDX models were generated. The study showed a varying resemblance between the primary tumor and derived models, with up to 80% discordance in the observed SNVs and indels, which was suggested to be explained by ITH. For most patients, 20–40% discordance between the sibling models was observed. Whether this was due to ITH or differences in selection pressure between models is unknown. In the present study, we found that each spheroid culture contained a mean of 17% private mutations. Consequently, by establishing spheroid cultures from multiple distinct tumor sites, a greater knowledge of the genetic landscape of the entire tumor is obtained. The heterogeneity of coding SNVs between spheroids from the same patient might cause phenotypic variation between cultures. Hence, establishing multiple spheroids per patient increases the representation of the different genetic subclones present in the primary tumor. Consequently, drug testing may become more accurate, as there will be a greater chance of discovering a potentially resistant clone, as exemplified by pt. 2 where the metastatic clone was only observed in one of three spheroid cultures. It is near impossible to ensure that all subclones are present in the cultures; hence, there is a potential risk of missing clinically important information which could lead to unsuccessful patient treatment. It should be taken into consideration that predominantly cancer cells grow in the spheroid cultures, lowering the stromal and immune components leading to a reduced representation of the tumor as such. Cancer cells in a primary tumor are highly influenced by the microenvironment including the stromal and immune cells, which might influence their drug sensitivity (Junttila and de Sauvage, 2013). The present spheroid model will only capture drugs that have a direct effect on the cancer cells. Such mechanisms are carried by most of the currently used therapeutic principles for the treatment of CRC. Coculturing of spheroids with stromal/immune cells may expand the chemosensitivity testing of drugs that, for example, target the immune system (Adjei and Blanka, 2015). Nevertheless, further studies are needed, particularly involving larger numbers of patients and preferably in parallel with spheroid drug screening, to fully establish the importance of ITH and the effect of multiple biopsies.

Transcriptional subclassification has recently been proven to resolve most of the intertumor heterogeneity observed in CRC, thereby enabling improved strategies for biomarker development (Bramsen *et al*., 2017). Here, we wanted to investigate how genetic ITH influences the precision of both tumor and cancer cell subclassification. Bearing in mind the considerable genetic ITH, we observed that the tumor subtypes (TUMOR and CMS) were surprisingly stable, which contrasted the cancer cell subtypes (CRIS) that showed major variation. We speculate that this may reflect the CRIS subtypes and the genetic ITH both being measures of the cancer cells, while the tumor subtypes are more stable because they reflect both the epithelial and nonepithelial cells in the samples.

It has been reported that also tumor subtype classifiers may show a variation depending on sampling site (Dunne *et al*., 2016). However, this was primarily observed for biopsies collected at the tumor front where obviously the fraction of nonepithelial cells is likely to vary from biopsy to biopsy. Importantly, all biopsies used in this study were collected from the luminal side of the tumor and within the morphological border of the tumor, which may explain why we find the tumor subtype assignments robust and largely independently of biopsy site.

Accordingly, in our hands, one biopsy may in most cases be sufficient for TUMOR and CMS subtype classification, although three or more biopsies would provide a more robust subtype. CRIS classification, on the other hand, calls for multiple biopsies per tumor. Further studies are needed to determine the appropriate number of biopsies and biopsy sites.

## 5 Conclusion

In conclusion, this study provides insight into the advantages of sampling multiple areas of the primary CRC tumor and establishing spheroid cultures from each area. A single biopsy from a tumor is a random look into the components of the tumor and therefore varies depending on the sampling site. When establishing spheroids from a single tumor biopsy, only a fraction of the subclones from the tumor will be present simply due to spatial ITH. However, by sampling multiple distinct sites of the tumor, the representation of the tumor subclones will increase and thereby automatically heighten the insight into the properties of the tumor. Transcriptional tumor subtyping seems to be largely independent of genetic ITH and site of biopsy. This suggests that a single biopsy may be sufficient for tumor subclassification. By contrast, transcriptional cancer cell subtyping (by CRIS) appears to be heavily affected by genetic ITH and multiple biopsies will likely be required to get the full picture of the CRIS subtypes in a tumor.

## Author contributions

SSA, MJ, OT, JT, and CLA involved in study design. SSA, MJ, IN, and MRM acquired the data. SSA, PL, JBB, MK, SV, and CLA analyzed and interpreted the data. SSA and CLA drafted the manuscript. All authors critically revised the manuscript.

## Supporting information


**Fig. S1.** CNAs across all chromosomes for patient 5.

**Fig. S2.** RNA sequencing data for exon two of the RPTOR gene.

**Table S1.** Patient information.

**Table S2.** Sample overview.
