# Supplementary figures and images for: Characterization of genetic intratumor heterogeneity in colorectal cancer and matching patient‐derived spheroid cultures
Source: Mol Oncol. 2017 Nov 27;12(1):132–47. doi: 10.1002/1878-0261.12156 (PMC5748486; doi:10.1002/1878-0261.12156)

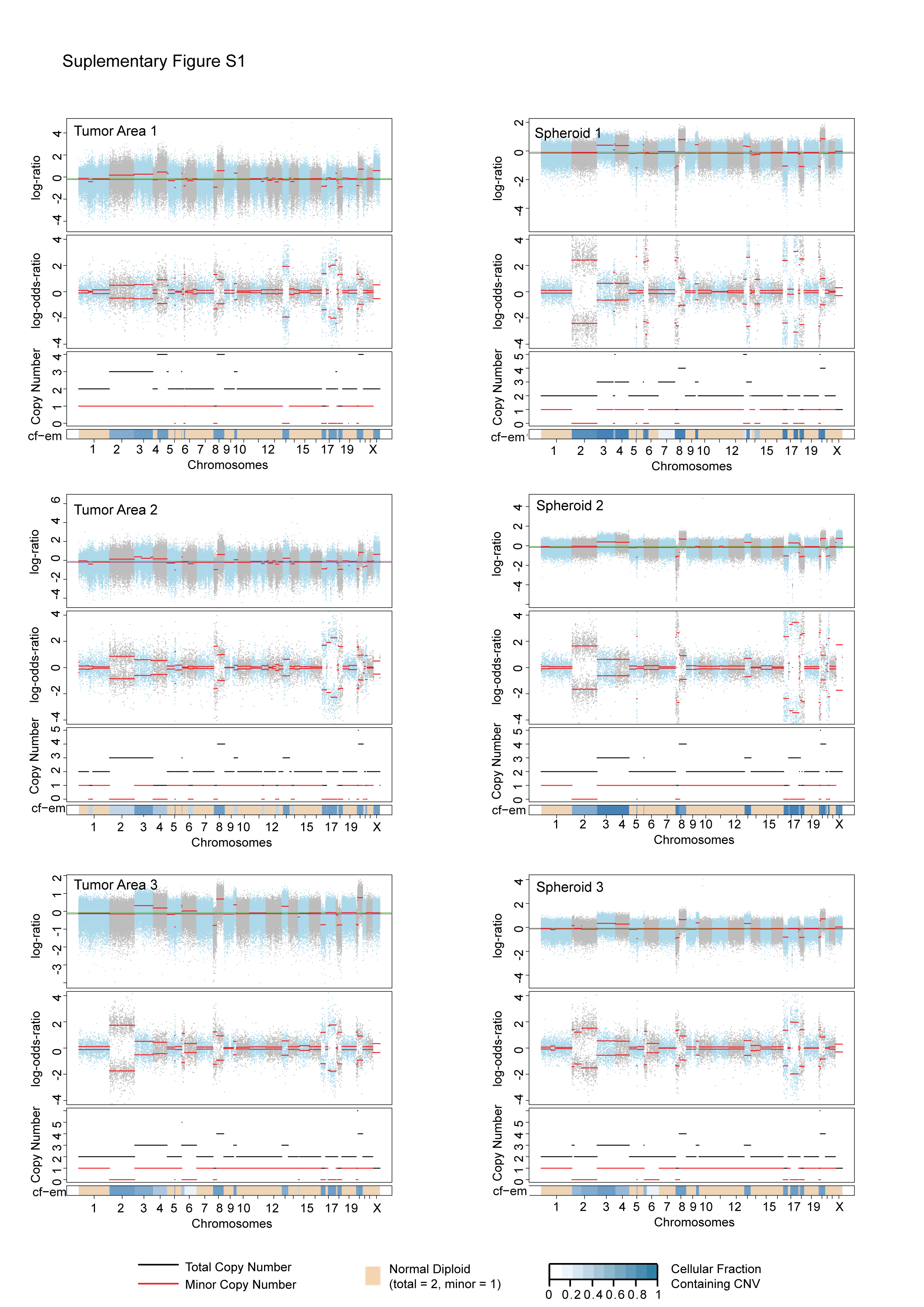

Supplement: Supplementary file 1 — Fig. S1. CNAs across all chromosomes for patient 5. [file MOL2-12-132-s001.tif]

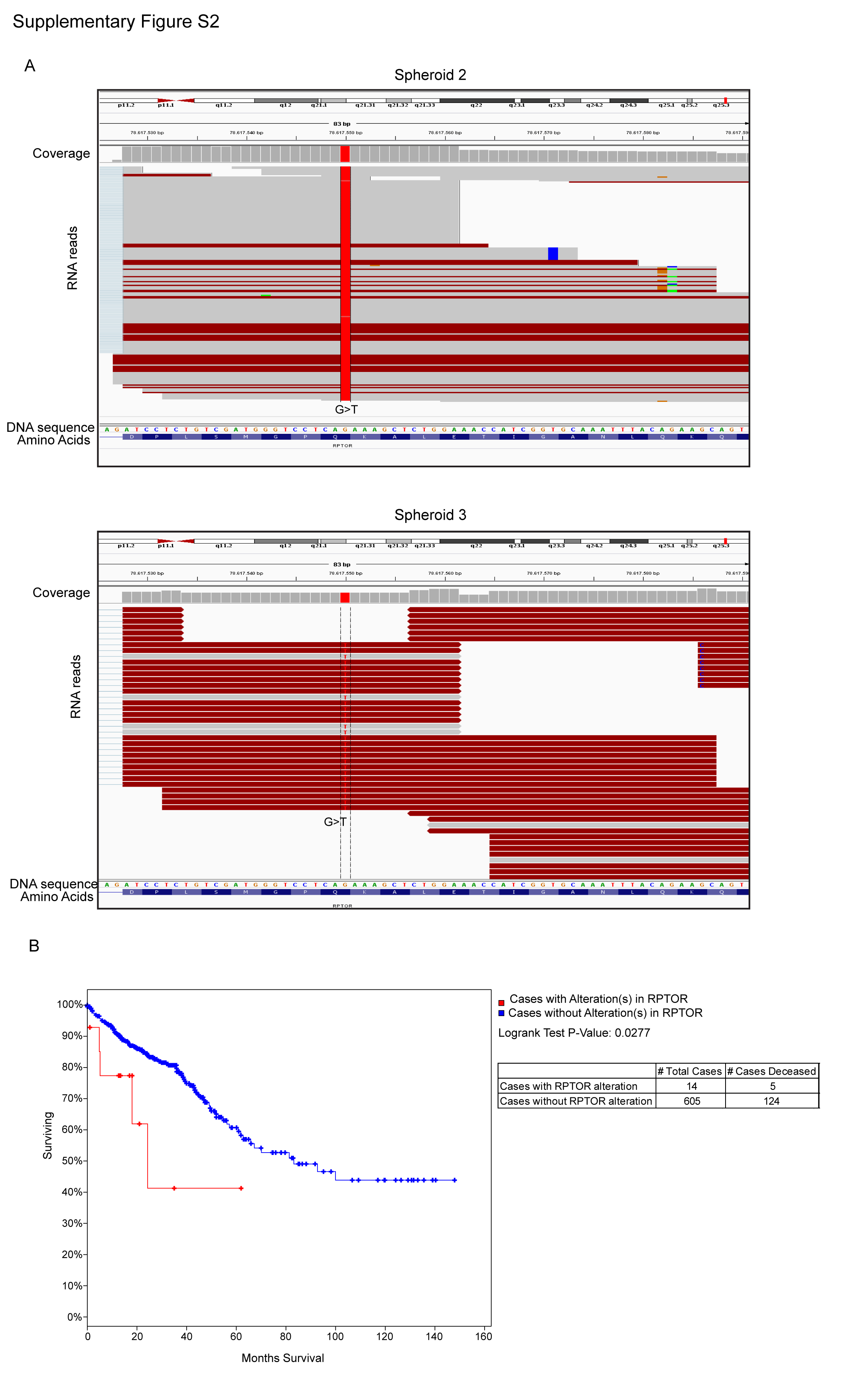

Supplement: Supplementary file 2 — Fig. S2. RNA sequencing data for exon two of the RPTOR gene. [file MOL2-12-132-s002.tif]
