# Supplementary material for: Characterization of genetic intratumor heterogeneity in colorectal cancer and matching patient‐derived spheroid cultures
Source: Mol Oncol. 2017 Nov 27;12(1):132–47. doi: 10.1002/1878-0261.12156 (PMC5748486; doi:10.1002/1878-0261.12156)
Supplement: Supplementary file 5 [file MOL2-12-132-s005.docx]

**Supplementary Figure S1** - CNAs across all chromosomes for patient 5. Some CNAs common for CIN CRCs are present in all samples e.g. loss of chr.8p and gain of chr.8q (containing *MYC*) (Cancer Genome Atlas, 2012). Other CNAs varies between samples. One example is chr.2, where there is LOH in all samples except T1. A second example is chr.X, which in normal conditions are 1n for the male patient 5. The mutated driver RBM10 is located on chr.X, and samples T1, T2, and S2 all have very high AFs for RBM10mut (as seen in Figure 1F). At the same time, they have a gain of the X chromosome, indicating that the mutation happened prior to the gain. S1 only have one X chromosome, but a high AF for RBM10mut, suggesting that the majority of cells in the spheroid cultures have the mutation. While T3 and S3 have lower AFs for RBM10mut, but only one X chromosome, indicating a mixture of clones with and without the mutation.

**Supplementary Figure S2** - RNA sequencing data for exon two of the RPTOR gene. **(A)** RNA sequencing data depicted in Integrative Genomics Viewer (IGV) (Robinson et al., 2011; Thorvaldsdóttir et al., 2013), for two spheroid cultures from patient 5. Both cultures exclusively expressed the mutated RPTOR containing a missense C>T transition. Spheroid 2 in 150 reads and Spheroid 3 in 29 reads. **(B)** Kaplan-Meier Plot for Overall Survival of CRC patients with or without mutations in RPTOR. From cBioPortal.org (Cerami et al., 2012; Gao et al., 2013) using a provisional TCGA dataset (n=633) (<http://bit.ly/2tbMjVk>). Mutations in RPTOR on either SNV or CNA level lead to significantly lower survival (p=0.0277, Logrank Test).

**Supplementary Table S1 –** Patient information. Patient ID, Number of biopsies, spheroids, and LNMs analyzed. Tumor localization, Tumor Stage, Patient age at surgery, Gender (male/female), Mismatch repair status, and whether WES and/or RNA sequencing was performed.

**Supplementary Table S2 –** Sample overview. Overview of all samples incl. sample IDs with indication of which samples were used for WES and RNA-sequencing. “Sample Type” indicates the origin of DNA/RNA, while “Sample” indicates tumor/spheroid/LNM area.

**Supplementary References**

Cancer Genome Atlas, N., 2012. Comprehensive molecular characterization of human colon and rectal cancer. Nature 487, 330-337.

Robinson, J.T., Thorvaldsdottir, H., Winckler, W., Guttman, M., Lander, E.S., Getz, G., Mesirov, J.P., 2011. Integrative genomics viewer. Nat Biotech 29, 24-26.

Thorvaldsdóttir, H., Robinson, J.T., Mesirov, J.P., 2013. Integrative Genomics Viewer (IGV): high-performance genomics data visualization and exploration. Briefings in Bioinformatics 14, 178-192.
